# Supplementary material for: GPBSO: Gene Pool-Based Brain Storm Optimization for SNP Epistasis Detection
Source: Genes (Basel). 2025 Sep 19;16(9):1114. doi: 10.3390/genes16091114 (PMC12469498; doi:10.3390/genes16091114)
Supplement: Supplementary file 1 [file genes-16-01114-s001.zip › Fig/genes-3838833- FigS1-12.pdf]

genes

MDPI

Article

GPBSO: Gene Pool-Based Brain Storm Optimization for SNP Epistasis Detection

Liyan Sun [1], Yi Xin [1,*], Shen Qu [2], Linxuan Zheng [1] and Linqing Jiang [1]

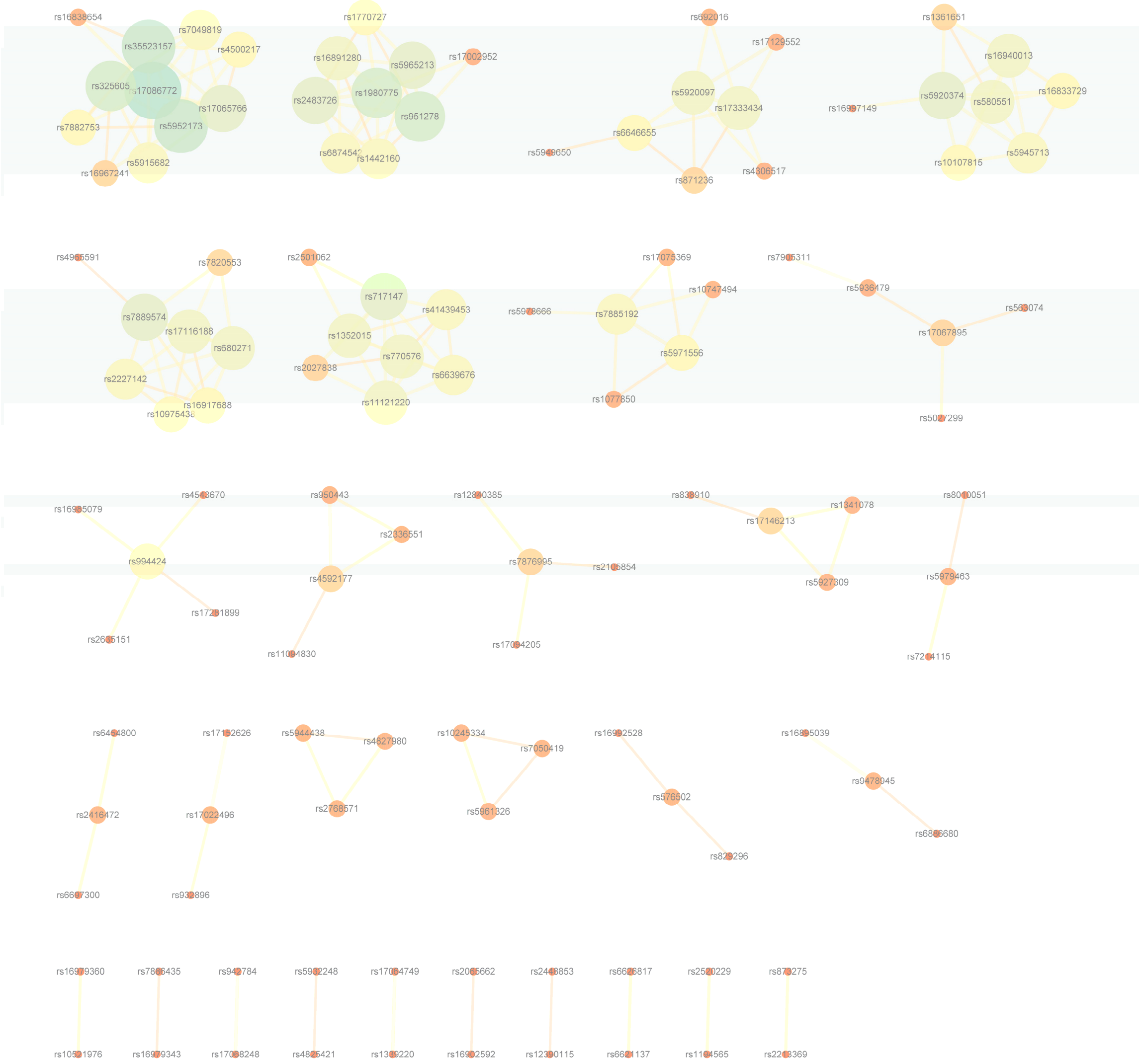

Figure S1. SNP-level interaction networks for six WTCCC diseases Coronary Artery Disease, constructed from high-confidence SNP pairs with the lowest G-test p-values after frequency filtering

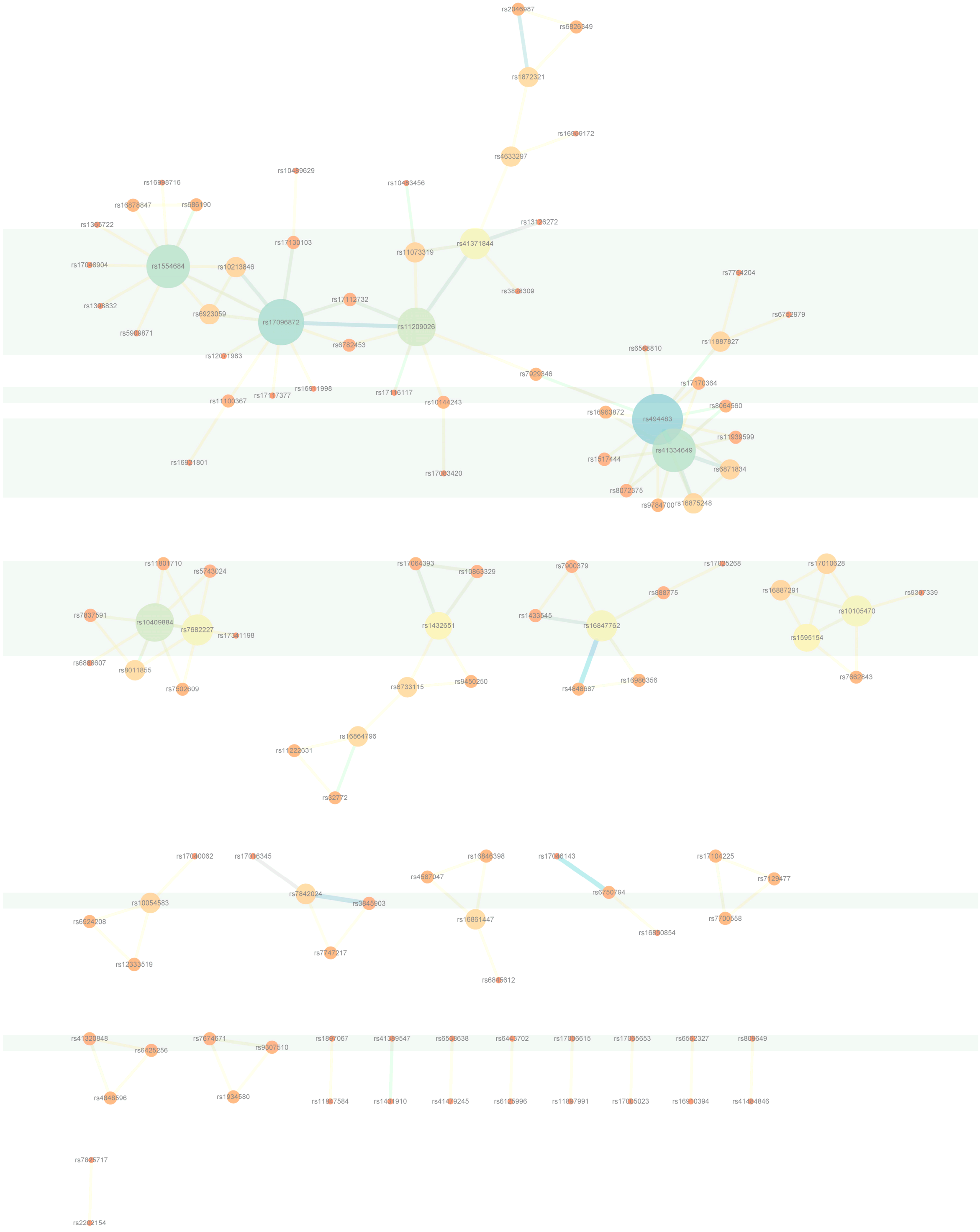

Figure S2. SNP-level interaction networks for six WTCCC diseases Crohn's Disease, constructed from high-confidence SNP pairs with the lowest G-test p-values after frequency filtering

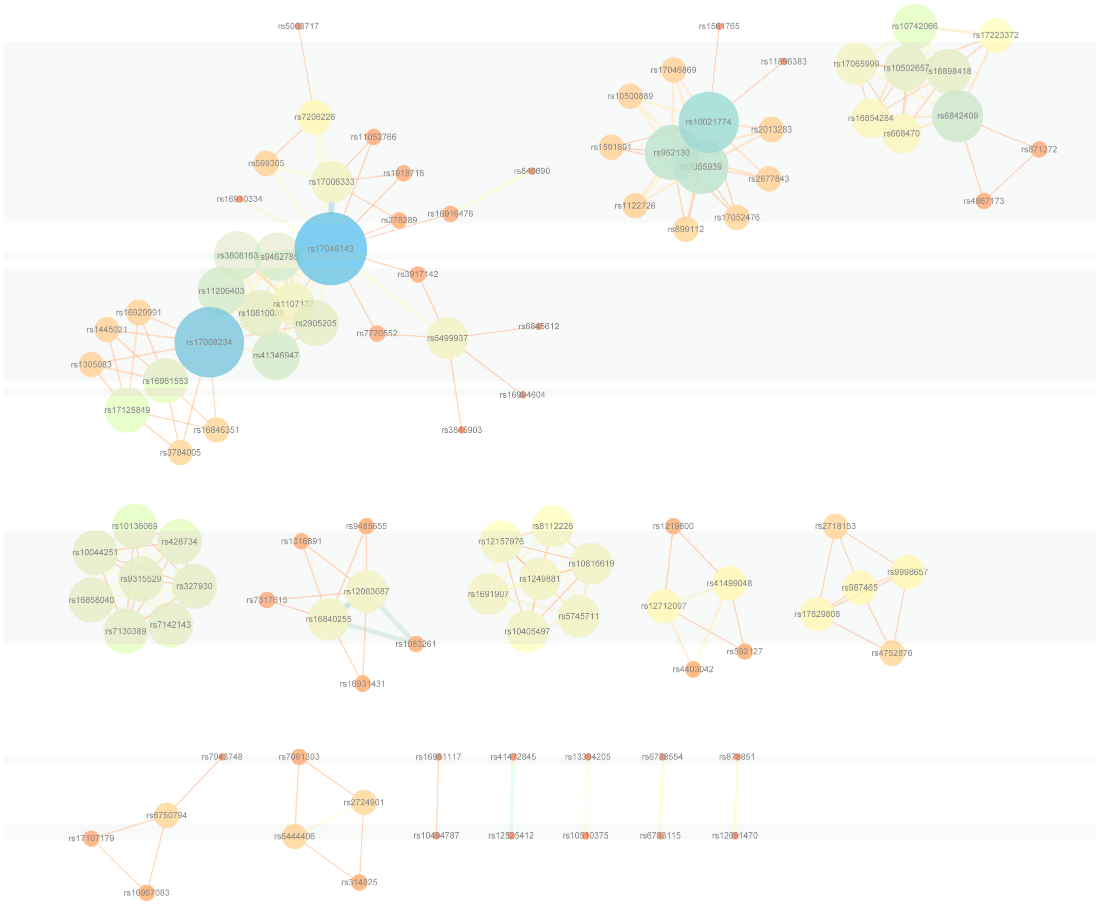

Figure S3. SNP-level interaction networks for six WTCCC diseases Hypertension, constructed from high-confidence SNP pairs with the lowest G-test p-values after frequency filtering

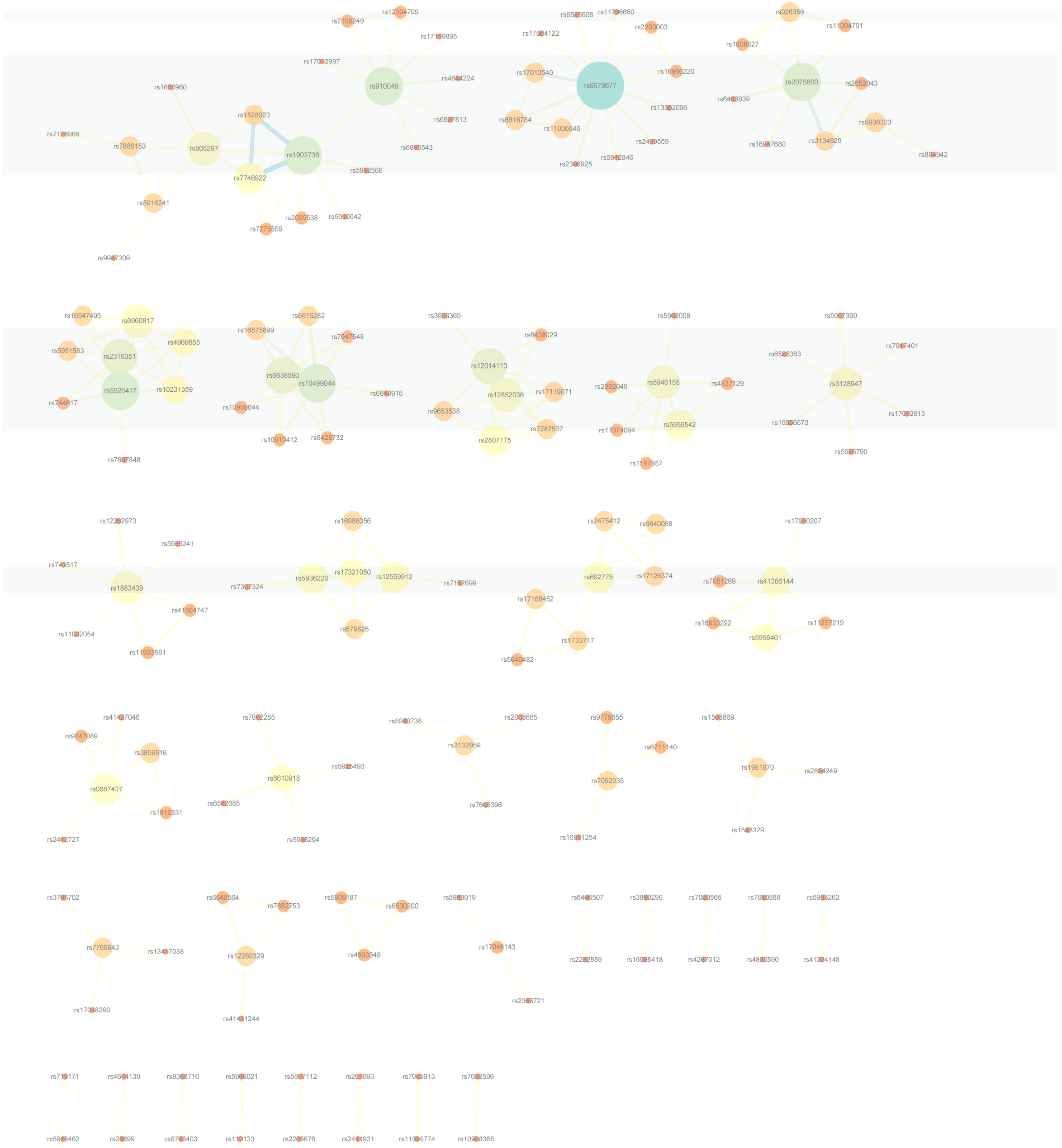

Figure S4. SNP-level interaction networks for six WTCCC diseases Rheumatoid Arthritis, constructed from high-confidence SNP pairs with the lowest G-test p-values after frequency filtering

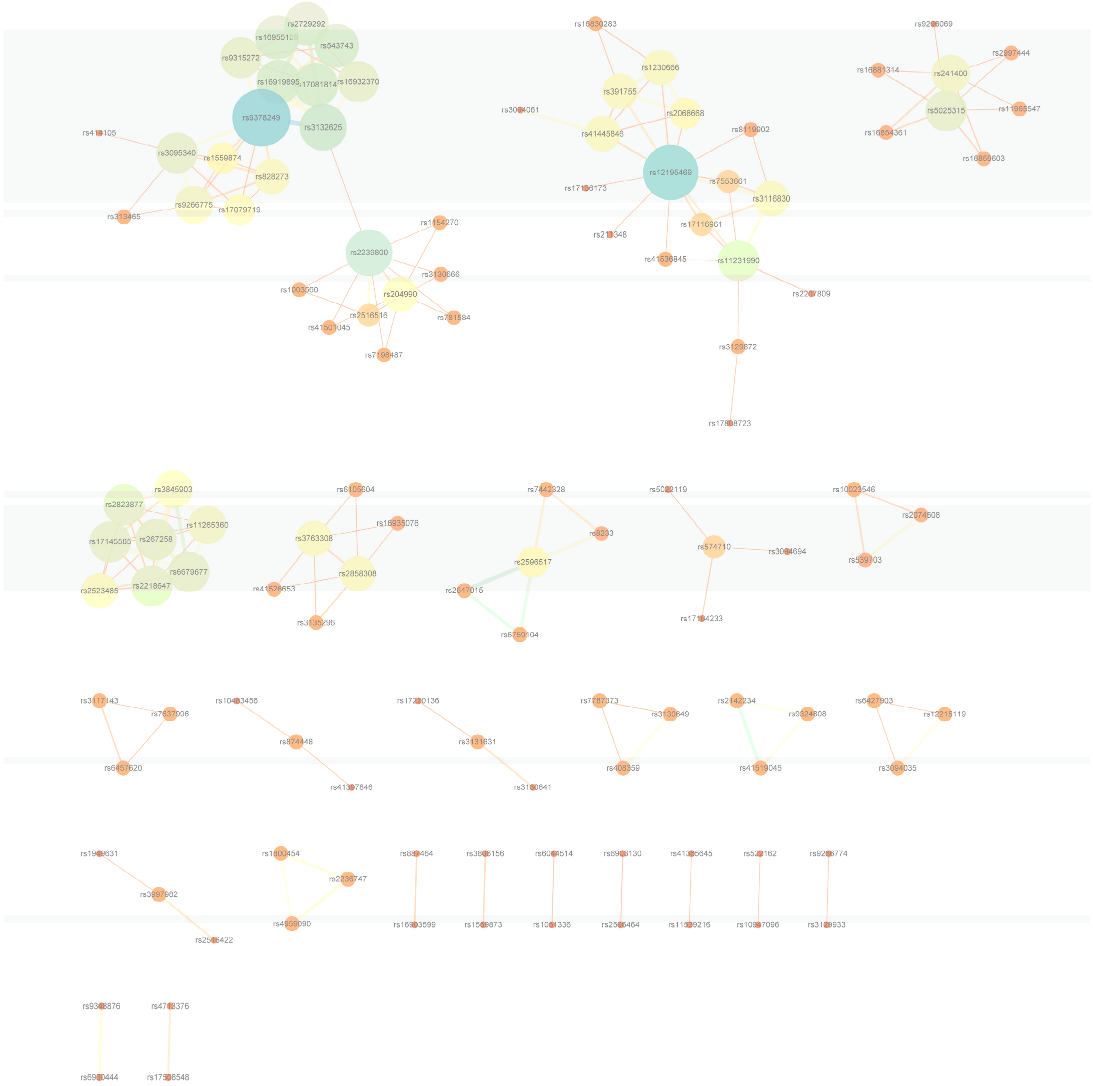

Figure S5. SNP-level interaction networks for six WTCCC diseases Type 1 Diabetes, constructed from high-confidence SNP pairs with the lowest G-test p-values after frequency filtering

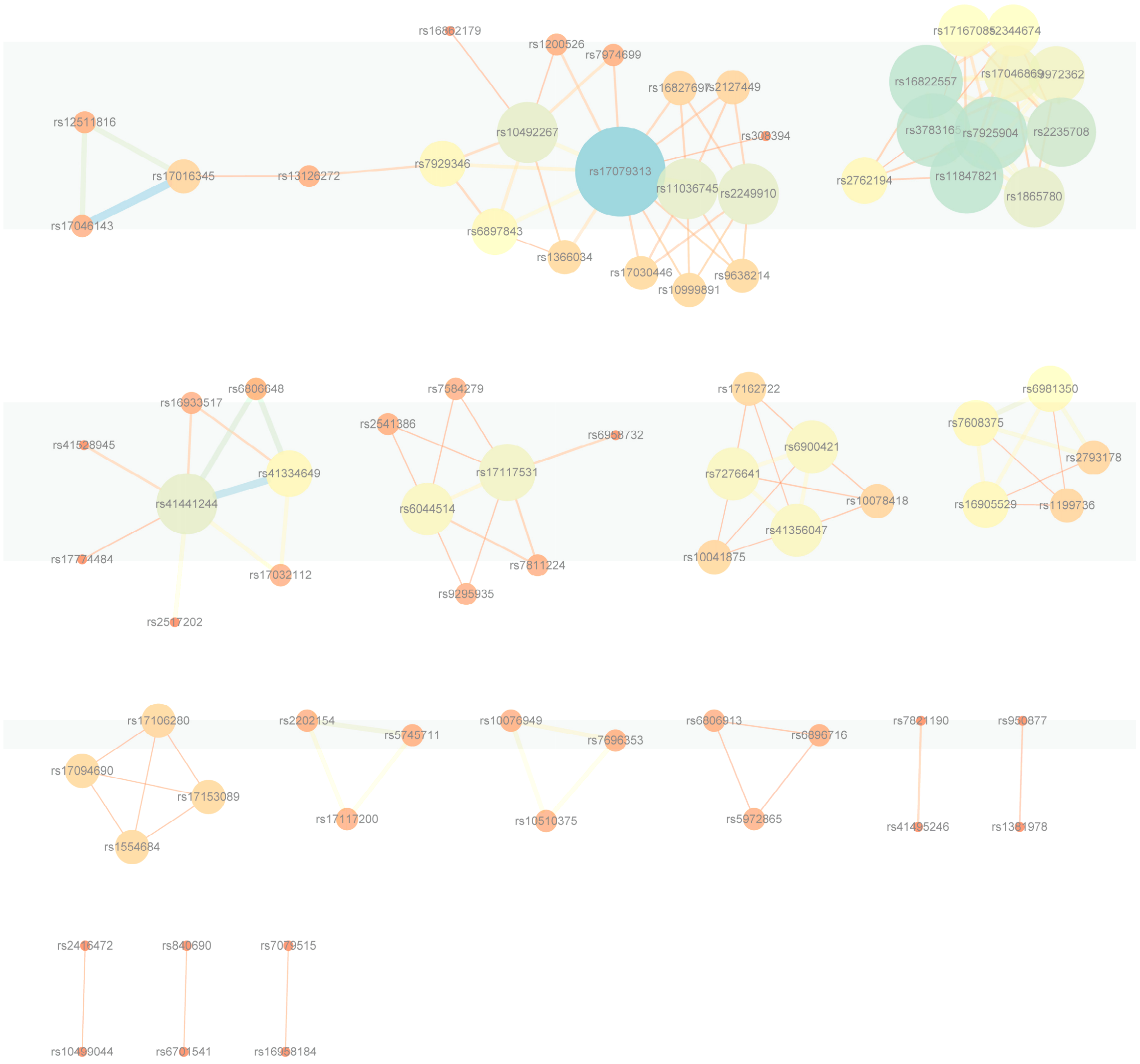

Figure S6. SNP-level interaction networks for six WTCCC diseases Type 2 Diabetes, constructed from high-confidence SNP pairs with the lowest G-test p-values after frequency filtering

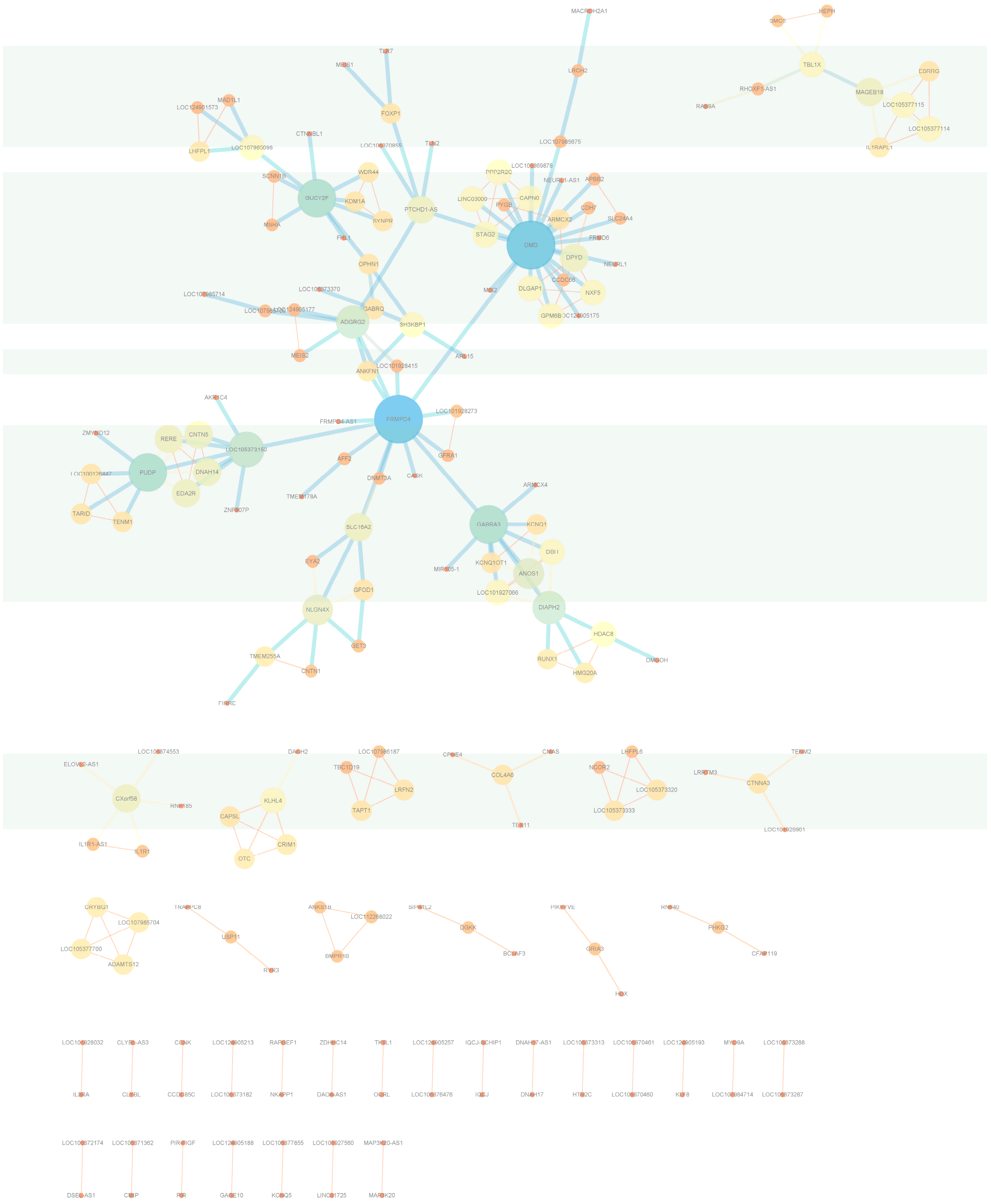

Figure S7. Gene-level interaction networks for the same six WTCCC diseases Coronary Artery Disease, constructed from high-confidence gene pairs with the highest co-occurrence frequency after frequency filtering.

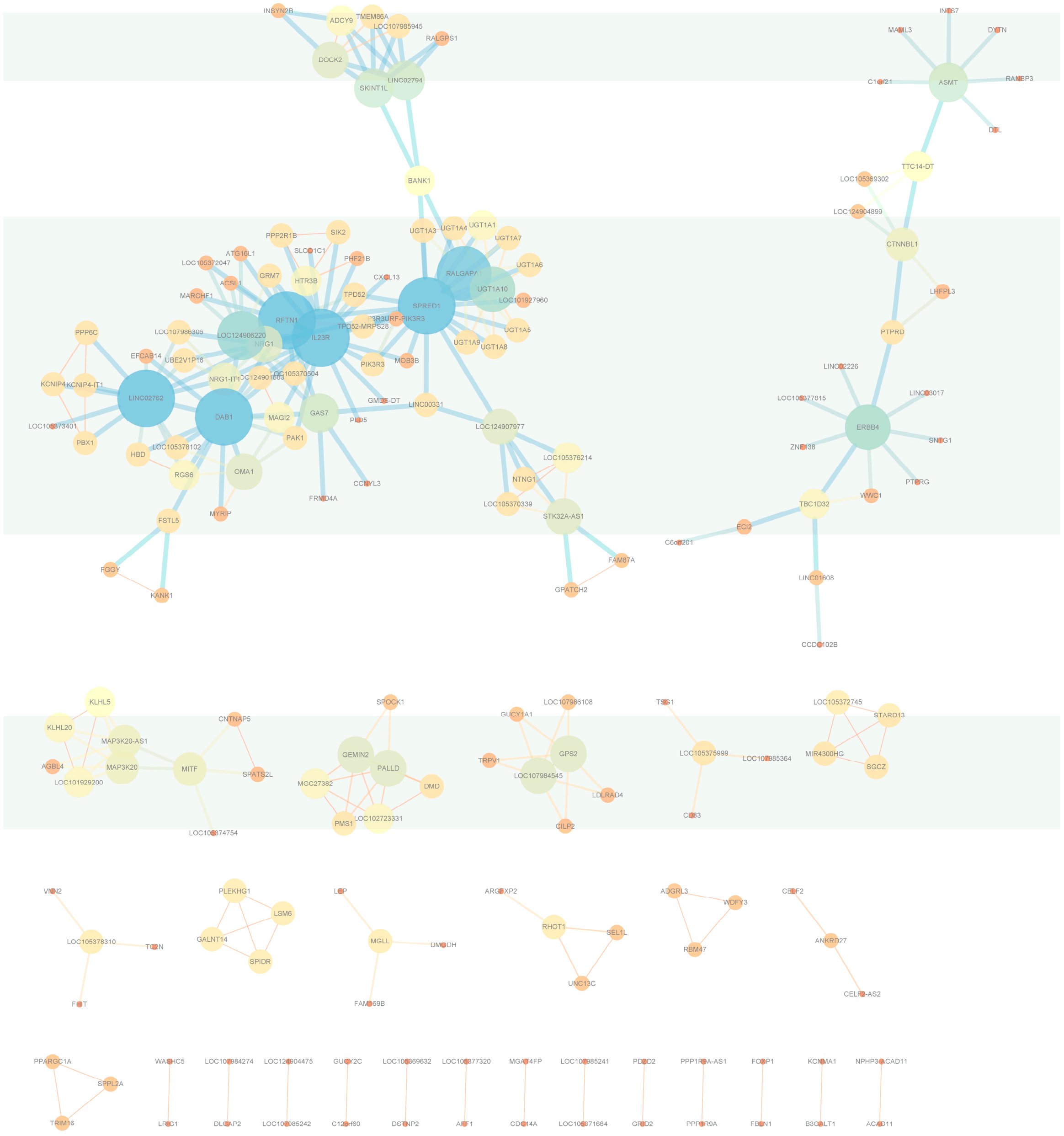

Figure S8. Gene-level interaction networks for the same six WTCCC diseases Crohn's Dis-ease, constructed from high-confidence gene pairs with the highest co-occurrence frequency after frequency filtering.

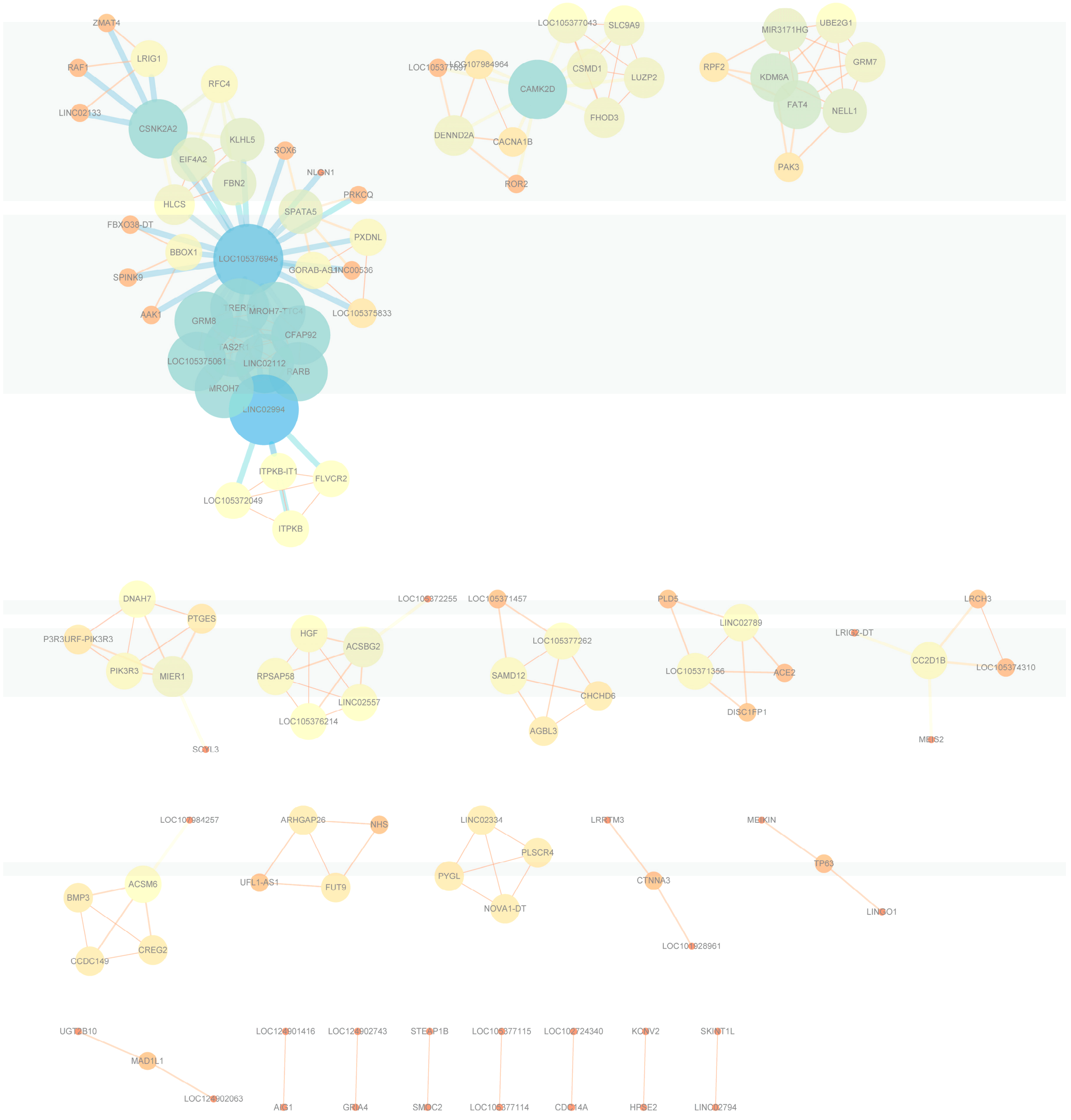

Figure S9. Gene-level interaction networks for the same six WTCCC diseases Hypertension, constructed from high-confidence gene pairs with the highest co-occurrence frequency after frequency filtering.

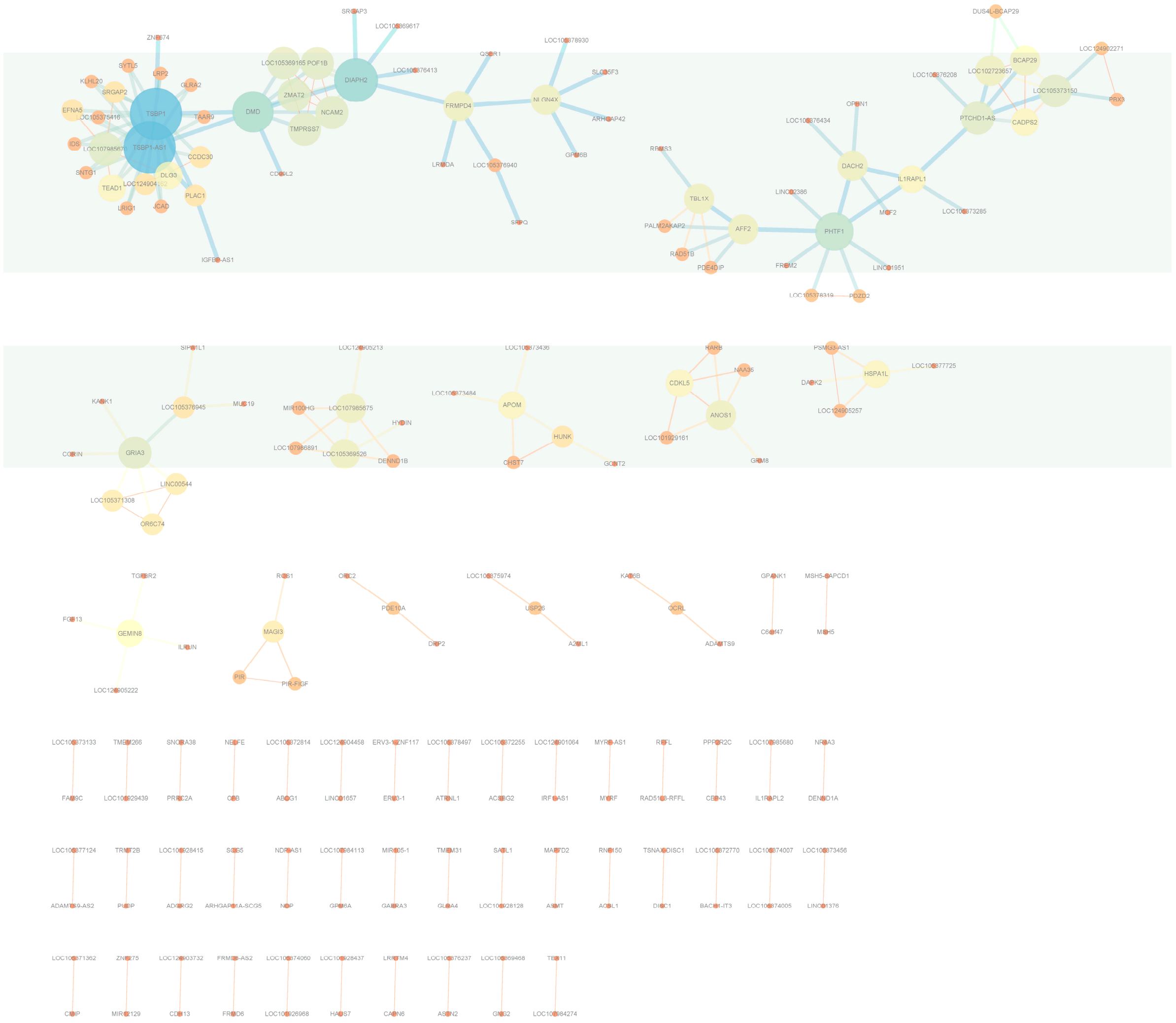

Figure S10. Gene-level interaction networks for the same six WTCCC Rheumatoid Arthritis, constructed from high-confidence gene pairs with the highest co-occurrence frequency after frequency filtering.

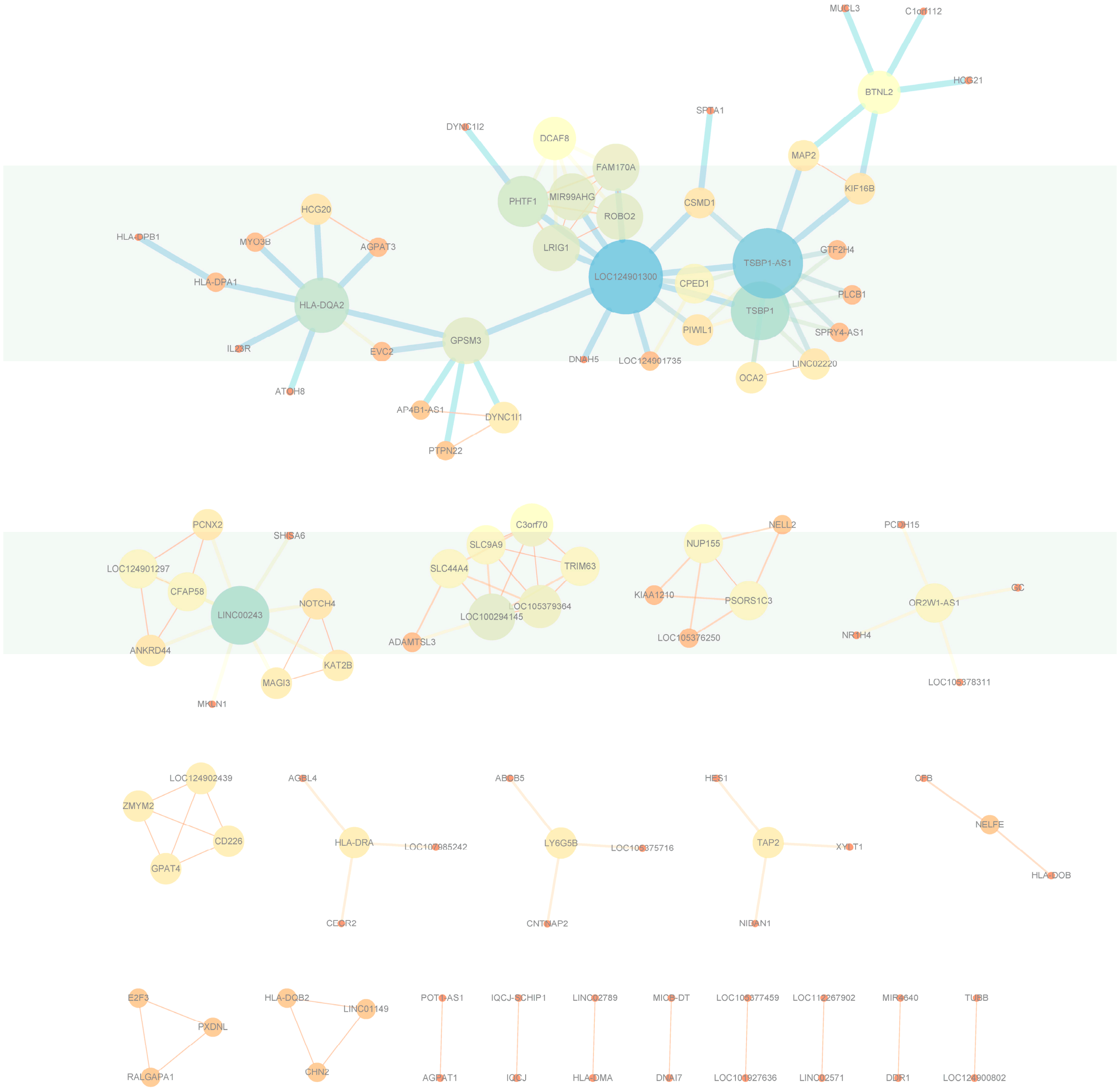

Figure S11. Gene-level interaction networks for the same six WTCCC diseases Type 1 Diabetes, constructed from high-confidence gene pairs with the highest co-occurrence frequency after frequency filtering.

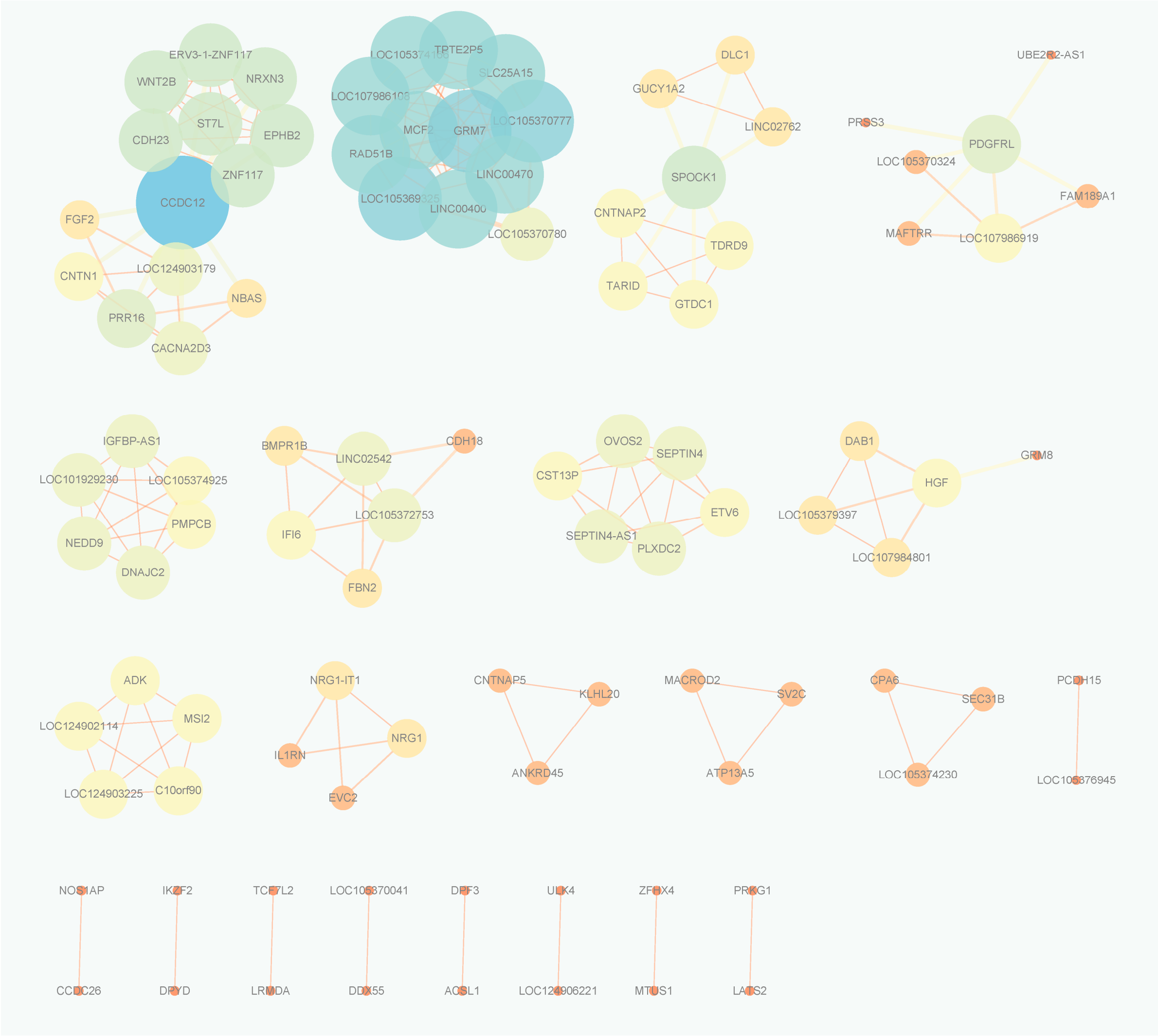

Figure S12. Gene-level interaction networks for the same six WTCCC diseases Type 2 Diabetes, constructed from high-confidence gene pairs with the highest co-occurrence frequency after frequency filtering.
