# Supplementary figures and images for: GPBSO: Gene Pool-Based Brain Storm Optimization for SNP Epistasis Detection
Source: Genes (Basel). 2025 Sep 19;16(9):1114. doi: 10.3390/genes16091114 (PMC12469498; doi:10.3390/genes16091114)

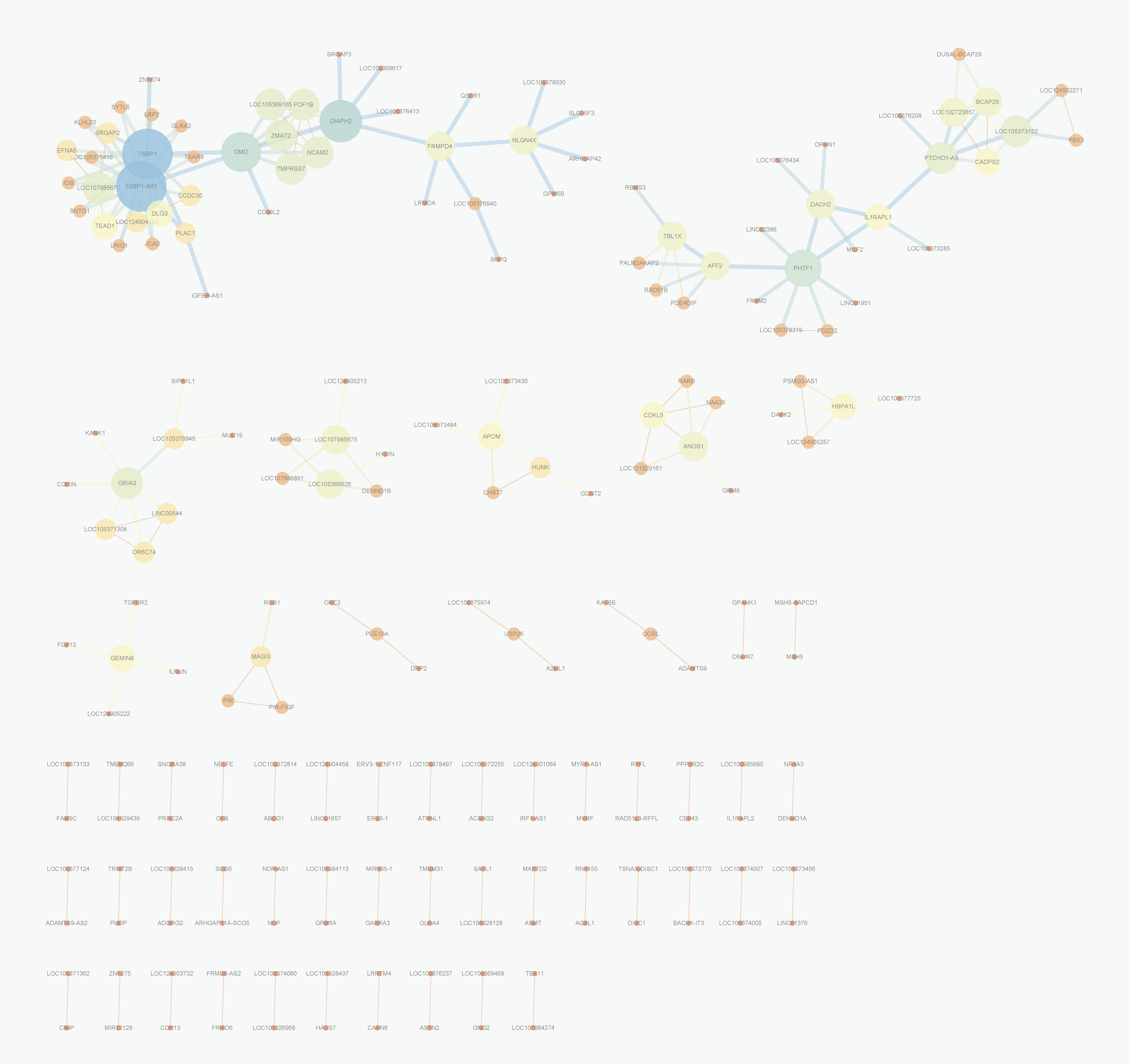

Supplement: Supplementary file 1 [file genes-16-01114-s001.zip › Fig/gene networks/S10.png]

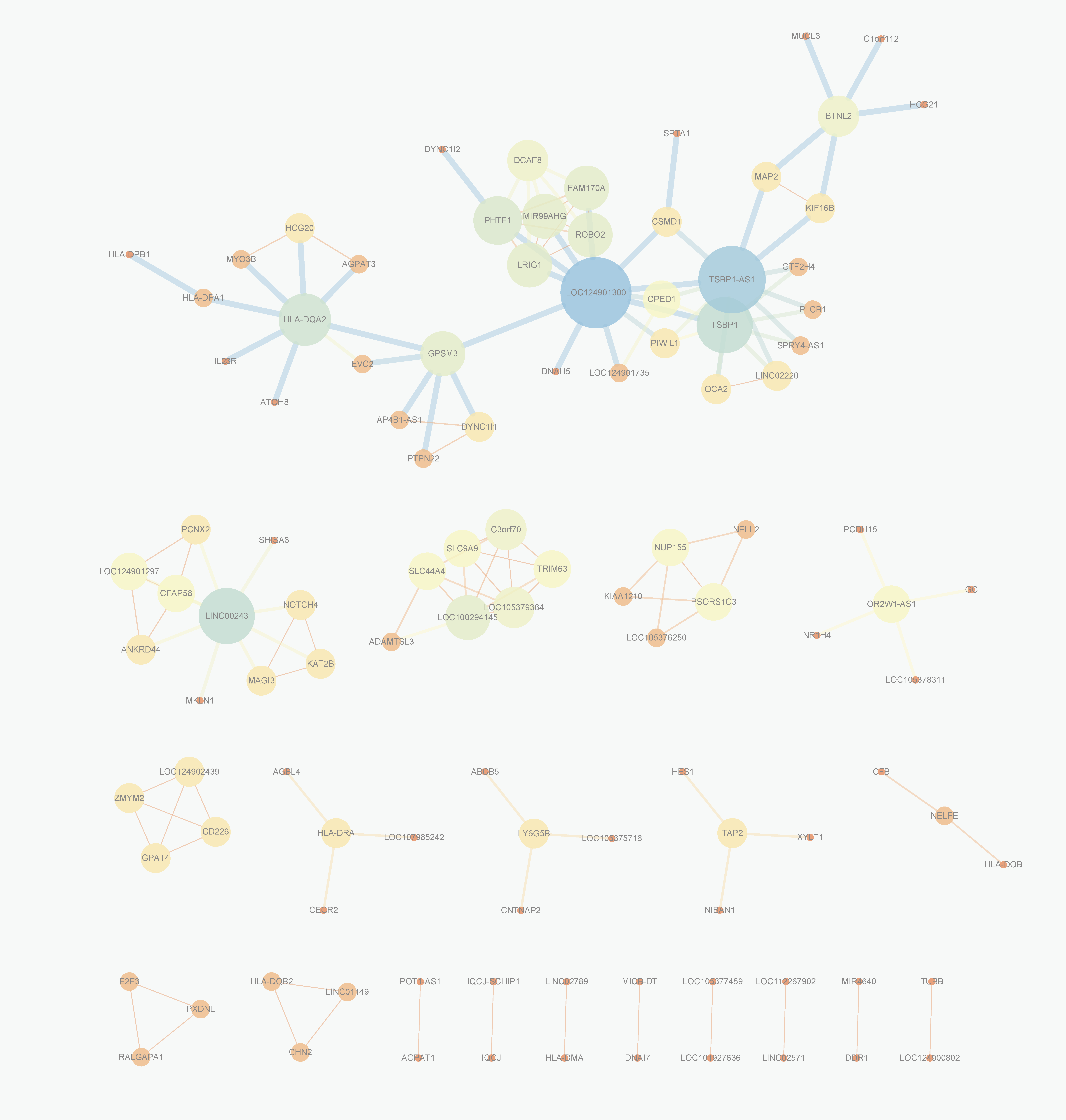

Supplement: Supplementary file 1 [file genes-16-01114-s001.zip › Fig/gene networks/S11.png]

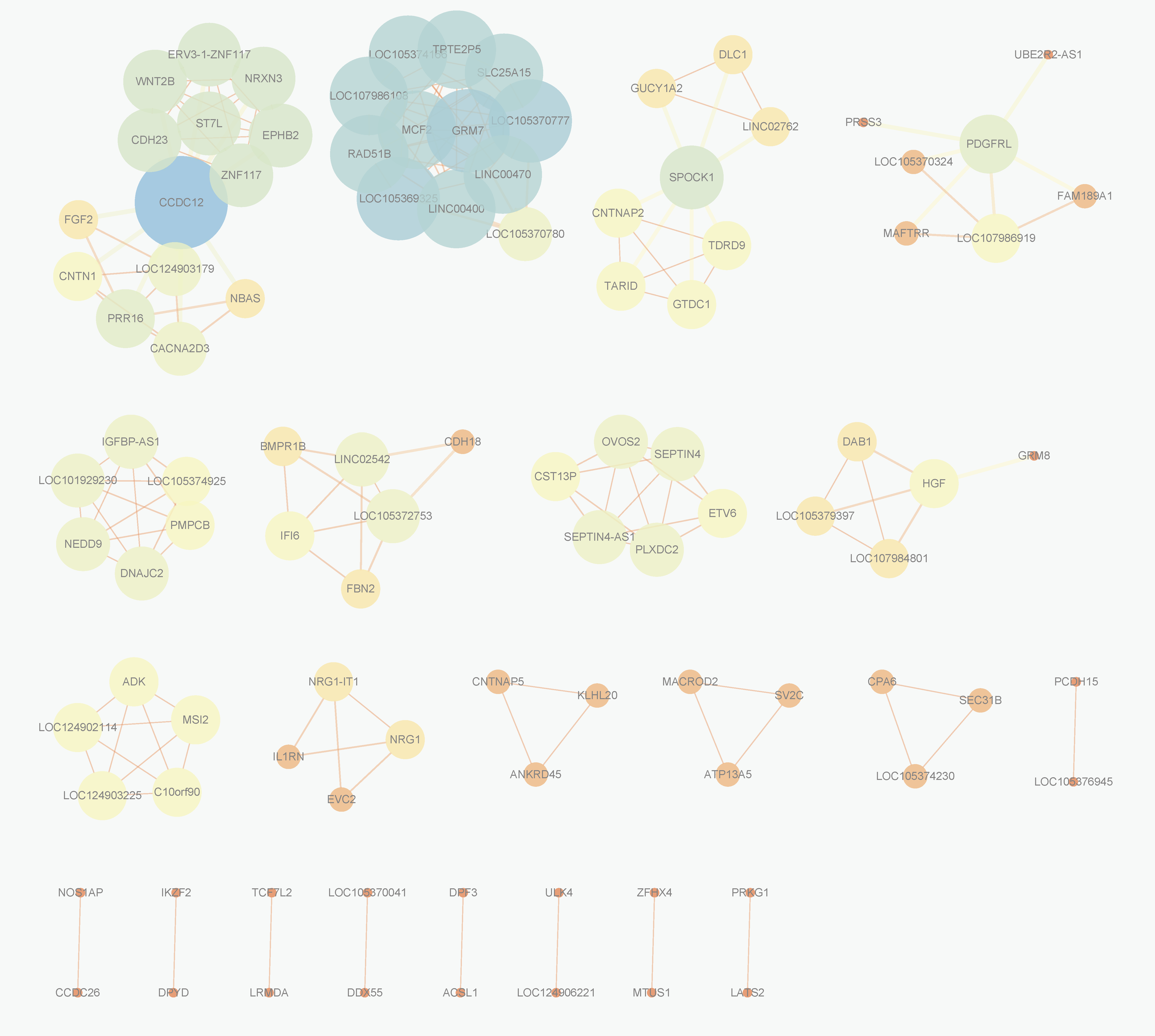

Supplement: Supplementary file 1 [file genes-16-01114-s001.zip › Fig/gene networks/S12.png]

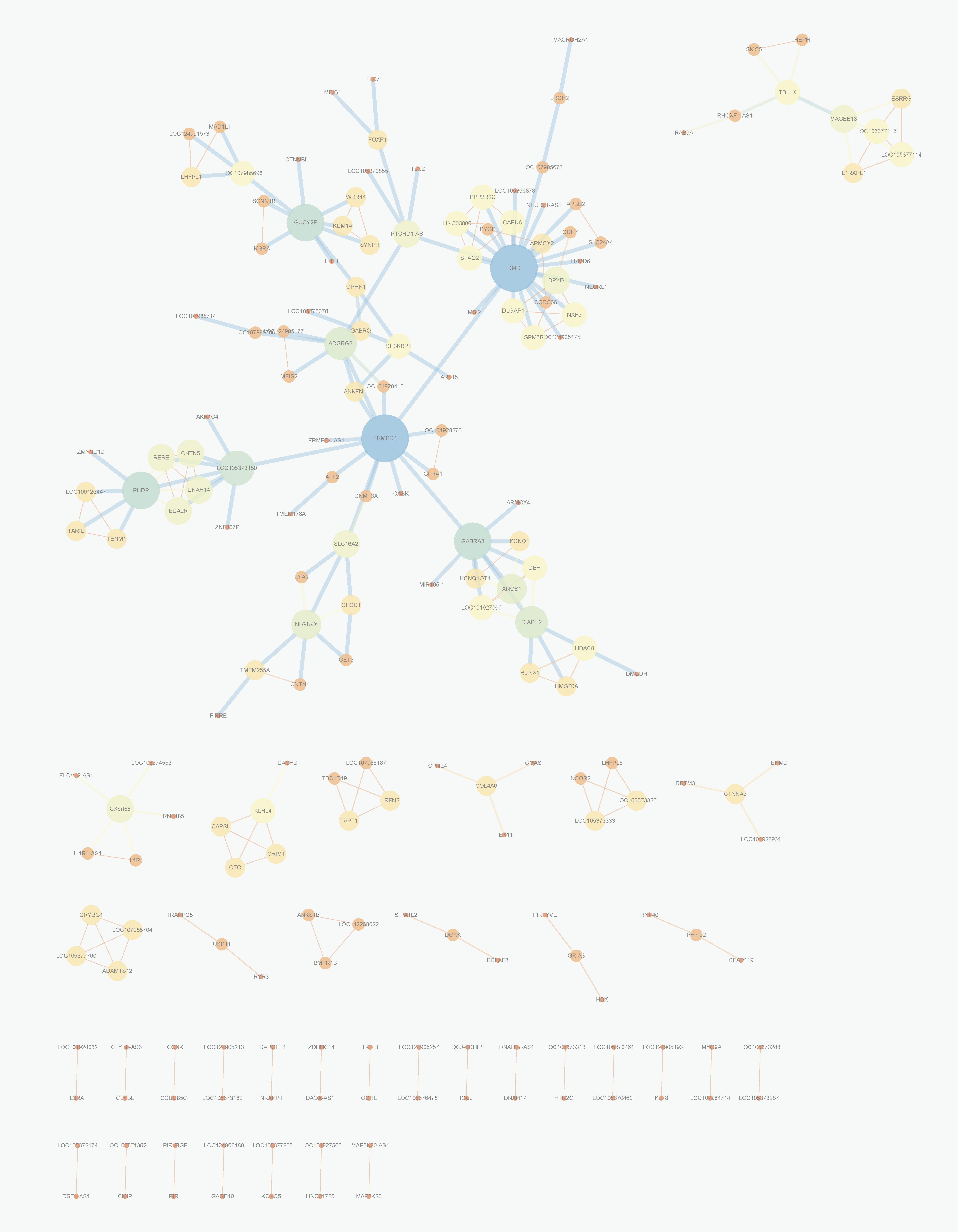

Supplement: Supplementary file 1 [file genes-16-01114-s001.zip › Fig/gene networks/S7.png]

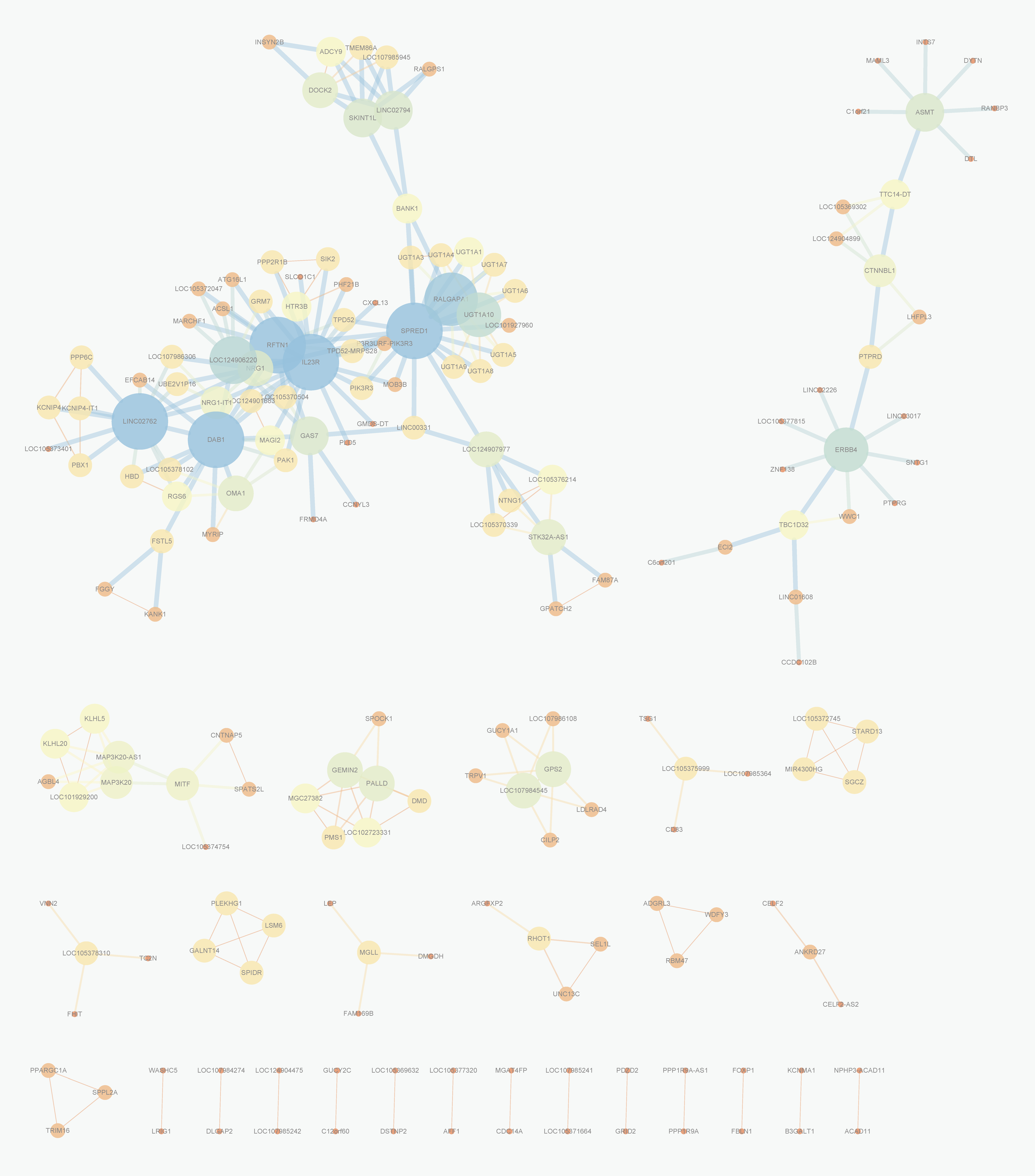

Supplement: Supplementary file 1 [file genes-16-01114-s001.zip › Fig/gene networks/S8.png]
